# Adverse Drug Reactions (ADR) in the inPatients of Medicine Department of a Rural Tertiary Care Teaching Hospital and Influence of Pharmacovigilance in Reporting ADR

**DOI:** 10.4103/0253-7613.40488

**Published:** 2008

**Authors:** A.P. Gor, S.V. Desai

**Affiliations:** Department of Pharmacology, Pramukhswami Medical College, Karamsad (GUJARAT) - 388 325

**Keywords:** Adverse drug reactions, ADR monitoring, Pharmacovigilance

## Abstract

**Objectives:**

(i) To find the incidence and study various aspects of Adverse Drug Reactions (ADR) in the inpatients of medicine department of Shree Krishna Hospital, a rural tertiary care teaching hospital. (ii) To test the impact of pharmacovigilance in reporting ADR.

**Material & Methods:**

A prospective study involving 600 patients admitted to the medical wards and TB & Chest diseases ward over a period of six months and a retrospective analysis of 600 case files for the corresponding period of the previous year were carried out to find the incidence rate of ADR, study various aspects of ADR like causality assessment, drugs frequently causing ADR etc. Suitably structured and pre-tested format was used for compiling the data.

**Results:**

In the prospective study, 18 of the 600 patients (3%) developed ADR. A significant number (77.78%) of patients developed ADR between the 3^rd^ and 10^th^ days of administering the drug/s. As the number of drugs increased, the incidence of ADR also increased. Majority of ADR (72.22%) occurred due to chemotherapeutic agents. 66.67% of ADR involved the gastrointestinal tract. None of the ADR was fatal. Sex of the patients did not influence the incidence rate of ADR. On the other hand, in the retrospective analysis, only ADR were reported in just 6 out of 600 patients (1%).

**Conclusion:**

The incidence rate of ADR is found to be much lower (3%) than the reported rate (10%-20%). Pharmacovigilance certainly contributes to picking up ADR.

Adverse drug reactions (ADR) are common occurrences in a hospital setting, attributed to the severity and complexity of the disease process, the use of multiple drugs, drug interactions and possible negligence.[1] ADR could be observed in 10-20% of hospitalised patients and may be responsible for prolonged hospital stay.[2] Moreover, a plethora of new drugs are now available, for which reporting of unexpected and rare ADR rests mainly on the alertness of the practising physicians.[3] Adverse drug reaction reporting directly adds to increased vigilance and may even influence the recommendations of drug use through regulatory authorities or pharmaceuticals alike.[4]

ADR could be monitored through active monitoring or through voluntary reporting system in a hospital set-up. Various reporting systems are operative in western countries, mostly in the form of spontaneous reporting systems, with or without obligatory legal bindings.[5]

It is not enough only to measure the incidence, nature and severity of adverse reactions to drugs, though accurate data are obviously useful.[6] Since there can be no hope of eliminating all adverse effects of drugs, it is necessary to evaluate patterns of adverse reaction against each other. One drug may frequently cause minor ill-effects but pose no threat to life. Some patients, e.g., those with a history of allergy or previous reactions to drugs, are up to four times more likely to have another adverse reaction, so that the incidence does not fall evenly.[7] Avoidable adverse effects will be reduced by more skilful prescribing and this means that doctors, amongst all the other claims on their time, must find time to understand drugs better, as well as to understand their patients and their diseases.[8] The study was undertaken to:

1. Find out the incidence, types and nature of ADR, causality drugs causing the same and their outcome amongst the hospitalised patients in the medicine ward of Shree Krishna Hospital (SKH) tertiary care teaching hospital located in the rural area and attached to Pramukhswami Medical College(PSMC), Karamsad.
2. Examine the impact of vigilance in estimating the extent of adverse reactions to drugs.

## Materials and Methods

A prospective study spread over 6 month's, duration, from July 2001 to December 2001, was carried out in the SKH Medical Research Center, for monitoring the ADR in the inpatients of medicine and TB & Chest Diseases departments. Simultaneously, a retrospective analysis of ADR in patients of medicine department, admitted between July 2000 and December 2000, was undertaken by examining the case files from the medical records office. Confidentiality of the information obtained was assured throughout the study.

Appropriate study protocol and proforma for monitoring ADR were developed and validated by a pilot study in 15 patients. Following patients were excluded: Patients not receiving drug therapy, patients referred by or transferred from other departments, patients discharged or transferred to other departments within 48 hours of admission and patients admitted to ICU.

All patients of either sex admitted in medical wards including TB and Chest ward during the study period and who did not fall in any of the above-mentioned categories were included in the study.

Information on all the patients including relevant history, examination details, investigations and drug therapy was collected and recorded in the above pre-tested proforma by visiting them daily till they were discharged from the hospital. When any other relevant information was required, the treating physicians were also contacted. Any ADR observed by the investigator or treating physician was noted in the proforma. Any untoward event was labelled as ADR only after the concurrence of the treating physician. In case of any difference of opinion with respect to reaction, treating physician's opinion was considered as final. To confirm adverse drug reactions, if some investigations were required, they were carried out with the consent of the concerned physician. In this study, only ADR were monitored. No observations / remarks were made on the diagnosis or management of the patient. The following information was noted down in separate columns in a register in a stratified manner.

**Table:** 

A: Patient information	B: Drug information:
1. Serial number	10. Day of starting administration of drug/s
2. Date of admission	
3. Date of discharge	11. Dosage form of drug/s
4. Age	12. Name/s of drug/s
5. Sex	13. Dose
6. Weight	14. Frequency of administration
7. Relevant investigations	
8. Diagnosis – provisional &final	15. Route of administration
9. Total duration of hospital stay	16. Duration of therapy
	17. Total number of drug/s
	18. Adverse drug reaction, if any
	19. Action taken and treatment of adverse drug reaction
	20. Outcome of management

Data were then further analysed to find out age and sex of the patients, incidence rate, drugs and body systems/organs involved, time of occurrence of adverse drug reactions, adverse drug reactions and total number of drugs administered, and effect of Pharmacovigilance on finding the incidence rate of adverse drug reaction. Data obtained by retrospective analysis were compared with the data collected by prospective study. Comparison of percentage of total adverse drug reactions during the two periods was made to find out any significant difference in the incidence rate.

Statistical Methods: The Chi-squared (X^2^) test was applied to find the association between the above parameters and p-values less than 0.05 were considered as significant.

## Results:

Out of 600 patients studied prospectively, 18 patients (3%) developed ADR (Table 1). Out of 18 patients developing ADR, 14 (77.78) developed within the first 10 days of administration of implicated drug.

**Table 1:** Incidence, causality and type of adverse drug reactions

*Characteristic*	*Male n (%)*	*Female n (%)*	*Total n (%)*
1. Incidence	09 (50)	09 (50)	18 (100)
2. Causality			
Definite	00 (0.00)	01 (05.55)	01 (05.55)
Probable	07 (38.88)	04 (22.22)	11 (61.11)
Possible	02 (11.11)	04 (22.22)	06 (33.33)
3. Reaction type			
A	09 (50)	09 (50)	18 (100)
B	00	00	0 (00)
4. Systems/organ			
Gastro-intestinal tract	7 (38.90)	5 (27.79)	12 (66.69)
Liver	-	3 (16.66)	3 (16.66)
Endocrine	1 (5.55)	-	1 (5.55)
Metabolic	1 (5.55)	-	1 (5.55)
Respiratory system	-	1 (5.55)	1 (5.55)
TOTAL	9 (50)	9 (50)	18 (100)

All 600 patients were divided into four groups according to the number of drugs received by them. The highest incidence of ADR (13.64%) was observed with the group receiving more than 10 drugs (Table 2).

**Table 2:** Number of drugs and adverse drug reaction

		*Number of patients n (%)*		
		
*Group of patients*	*No of drugs*	*ADR n (%)*	*No ADR n (%)*	*Total n (%)*
A	Upto 2	0(0.00)	16(100)	16(100)
B	3 to 5	7(2.14)	320(97.86)	327(100)
C	6 to 10	5(2.35)	208(97.65)	213(100)
D	> 10	6(8.64)	38(91.36)	44(100)
	Total	18(3.00)	582(97.00)	600(100)

ADR related to gastrointestinal tract were most frequent followed by liver and other organs (Table 1).Out of 18 ADR detected, the majority involved anti- infectives like chloroquine (5), amoxicillin (4) and antituberculosis drugs (4). In one case, the causative drug could not be identified (Table 3).

**Table 3:** Drugs causing ADR

*Sr. no.*	*Drug name*	*No. of patients receiving the drug*	*No. of patients developing ADR n (%)*
1	Amoxicillin	202	4(22.22)
2	Antitubercular	295	4(22.22)
3	Chloroquine	84	5(27.78)
4	6-mercaptopurine	01	1(5.56)
5	Enalapril	59	1(5.56)
6	Amiodarone	04	1(5.56)
7	Dexamethasone	28	1(5.56)
8	Alternative Medicine	01	1(5.56)
Total			18(100)

Further analysis based on number of patients receiving a particular drug revealed that 1 out of 1 patient, that is 100% of patients, receiving 6- mercaptopurine and 1 out of 4 (25%) patients receiving amiodarone developed ADR. On the same basis, incidence of ADR was lower for chloroquinine (5.95%), amoxicillin (1.989) and antitubercular drugs (1.369).

When causality assessment of 18 ADR was done, 1 belonged to definite category, while possible and probable ADR were 6 and 11, respectively. All 18 ADR were type A reactions (augmented) (Table 1).

In the retrospective study, only 6 out of 600 (1%) patients had documented ADR, compared to 3% in the prospective study.

## Discussion:

In the present study, the incidence rate of ADR was 3%. Various studies in the world have reported the incidence rate of ADR ranging from 6% to 20%. In a recent review, Pir Mohamed et al reported ADR frequencies between 10% and 20% in inpatients.[9] It was observed that for marketed drugs in the U.S., the ADR account for 3% to 7% of all hospitalizations. In prospective studies, ADR occurred in 10% to 20% of hospitalized patients, and 1% to 20% of the reactions were severe.[10] When compared with these studies, the incidence rate in our study (i.e., 3%) appears to be very low. In a recently conducted prospective study, spanning over four years for ADR monitoring, as a part of a multi-centric study of Indian Council of Medical Research, amongst the patients attending to medicine department of SSG hospital and medical college, Baroda, the incidence rate of ADR was found to be less than 3% (personal communication). Various factors may account for this apparently low rate of ADR. This may include genetic factors, ethnic factors, dietary and environmental factors etc. There is a need to explore the reasons for this relatively low incidence rate of ADR in the Indian population. However, it is also possible that the present study was carried out only in a small number of patients hospitalized in medicine department of a tertiary care teaching hospital. As is documented, most of the ADR develop within the first 10 days of administering the drug.[7] In our study, also significantly large number of patients (77.78%) developed ADR in the first 10 days. This emphasizes the need of observing the patients closely in the initial period of treatment. It is a well-established fact that as the number of drugs increases, the chance of developing ADR also increases.[12] In the present study, also we observed that as the number of drugs involved there was a significant increase in the rate of ADR. When the number of drugs was two or less then there was no ADR. On the other hand, the rate of ADR increased to 8.64% when more than 10 drugs were administered There was a significant association between the number of drugs and rate of ADR (*p* < 0.05). Antimicrobials top the list of the drugs causing ADR.(5) We also observed that 13 out of 18 (72.22%) patients in our study had ADR caused by chloroquine, amoxicillin and antitubercular drugs. Our study also revealed that incidence of ADR amongst patients receiving 6-mercaptopurine and amiodarone was very large, i.e., 100% and 25%, respectively. This is not surprising considering the fact that both these drugs belong to the categories of drugs having low therapeutic index. One of the objectives of the present study was to examine the effect of pharmacovigilance on finding the incidence rate of ADR. The prospective part of the study showed the incidence rate of ADR to be 3%. On the other hand, the incidence rate of ADR was found to be only 1% in the retrospective analysis. The difference between these two studies (retrospective and prospective) was significant (X^2^-6.12,*P* < 0.05). This clearly shows that when one looks for the occurrence of ADR consciously, more patients with ADR can be picked up. There is a need to inform the treating doctors about the importance of observing for ADR following pharmacotherapy, recording them meticulously and reporting them to the concerned authority. This practice will prove to be very valuable in making the drug therapy safer and rational. There was no influence of either age or sex on the occurrence rate of ADR in these patients. All these ADR were well-known reactions to the drugs concerned and none was a newly observed effect. All 18 reactions belonged to type A (augmented) reactions. As such, 80% of the reactions are of type A[10] and hence our finding is not surprising. A majority of the patients (61.11%) developed the ADR graded as “probable in nature”, since in all these cases the time sequence from taking the drug was reasonable; events corresponded to what is known of the drug; events ceased on stopping the drug and the events were not reasonably explained by the patient's disease. In 33.33% of the cases of adverse drug reactions, they were designated as “possible in nature” since the time sequence was reasonable, the events corresponded to what is known of the drug and the events could readily have been a result of the patient's disease or therapy. The only patient who was designated to be having an ADR of “definite” grade had developed cough following the administration of enalapril. As is expected, 12 out of 18 (66.67%) patients, who developed ADR, had symptoms relating to the gastrointestinal tract. This was followed by involvement of liver in 3 (16.66%) patients. While managing the patients of ADR, the first principle which is followed is to discontinue the suspected offending drug and replace the same by another drug if required. In our study, the drugs suspected to be causing ADR were discontinued in 12 of the 18 (66.67%) patients. In another four patients (22.22%), the offending drug was replaced by another suitable drug. It was noteworthy that no patient died as a sequel of ADR. All the patients except one (94.44%) recovered fully after discontinuing the offending drug. The only patient who developed hypothyroidism due to amiodarone was also receiving thyroxin concurrently. In this patient, amiodarone was stopped and the patient was discharged from the hospital, while continuing thyroxin therapy. This patient was not followed up.

Despite some limitations like short study limited to one department, our study has provided baseline data for further larger studies and has ascertained the importance of prospective ADR monitoring in pharmacovigilance studies.
